# Identifying Stigma Phenotypes in Social Media Narratives of Substance Use: Observational Study

**DOI:** 10.2196/68695

**Published:** 2025-11-13

**Authors:** Lexie Chenyue Wang, Kenneth C Pike, Mike Conway, Annie T Chen

**Affiliations:** 1 Department of Linguistics University of Washington Seattle, WA United States; 2 Office of Nursing Research University of Washington Seattle, WA United States; 3 School of Computing and Information Systems University of Melbourne Melbourne Australia; 4 Department of Biomedical Informatics and Medical Education School of Medicine University of Washington Seattle, WA United States

**Keywords:** stigma, substance use, social media, machine learning, social ecological

## Abstract

**Background:**

Individuals with substance use problems experience stigma in different contexts. Identifying characteristic situations in which stigma occurs or manifests—stigma phenotypes—can serve as important leverage points for future intervention.

**Objective:**

This paper aims to (1) identify stigma phenotypes expressed in social media narratives related to substance use stigma and (2) explore the similarities and differences between the stigma phenotypes from a social ecological perspective.

**Methods:**

We collected Reddit posts pertaining to 3 substances—alcohol, cannabis, and opioids. We performed feature engineering using a combination of content analysis, machine learning, and keyword-based methods to predict variables at different levels of the social ecological framework. Leveraging these features, we applied the fuzzy c-means clustering algorithm on the subset of posts containing stigma to extract stigma phenotypes, where a phenotype is defined by four main dimensions: (1) the stigma mechanism present (eg, internalized stigma, anticipated stigma, or enacted stigma), (2) the substance used (eg, alcohol, cannabis, or opioids), (3) the settings involved (eg, work, school, or home), and (4) the actors involved (eg, family, friends, or partners). Finally, we used Kruskal-Wallis and Dunn post hoc tests to examine the differences between stigma phenotypes with respect to specific ecological factors.

**Results:**

We derived 7 stigma phenotypes from stigma-related posts by 8627 authors. The phenotypes can be categorized into 4 groups: internalized stigma–only, anticipated stigma, enacted stigma–only, and mixed-stigma phenotypes. Narratives on internalized stigma phenotypes focused on the self, with minimal reference to settings and actors. One phenotype focused on anticipated stigma and was characterized by a high proportion of opioid use mentions (707/1217, 58.09% of the authors) and references to the health care setting (647/1217, 53.16% of the authors). Posts associated with the enacted stigma–only phenotypes included substantial representation of settings and actors. Narratives in the mixed-stigma phenotypes often involved more than one stigma mechanism, setting, and actor, with home and family being the most salient factors. The phenotypes differed from one another with respect to social ecological factors, including loneliness and social isolation, use of treatment services, presence of health care providers, community and support groups, society, and legalization.

**Conclusions:**

These findings provide valuable insights that help inform the design and development of interventions targeted at different stigma phenotypic groups from a social ecological perspective.

## Introduction

### Background

*Stigma*, a term originating in ancient Greece, was initially used to refer to physical marks on an individual’s body that signified their status as a criminal, slave, or traitor [1]. In contemporary times, the concept of stigma can include labeling, stereotyping, social exclusion, status loss, and discrimination against individuals [2]. People with substance use disorders frequently experience stigma, which can adversely impact different aspects of their lives, including physical and mental well-being, employment opportunities, social status, relationships, health care, and more [3,4]. Specifically, the negative impacts may include reduced social functioning [5], social disapproval and labeling [6], distrust in health care providers and reluctance to seek treatment [7,8], delayed recovery [9], and insufficient health care [9].

Understanding factors that shape substance use stigma experiences is necessary for effective intervention design for stigma reduction, substance use recovery, and improvement of overall mental and physical health. In this study, we focused on 3 groups of factors: substances, stigma mechanisms, and ecological factors. We focused on 3 substances—alcohol, cannabis, and opioids. Due to differences in legality, societal acceptance, and other considerations, we expected the nature of stigma-related experiences with each substance to differ.

We studied 3 stigma mechanisms—enacted, anticipated, and internalized stigma, as defined in the stigma framework [10,11]. Enacted stigma refers to direct experiences of stereotyping and discrimination enacted by another individual because of a stigmatized attribute [12]. One example is being called a “drug addict” by another person. Anticipated stigma is the expectation that others might hold stereotypical and discriminatory views toward the self [12], for example, hiding liquor bottles from family members due to fear of judgment. Internalized stigma, also known as self-stigma, is the experience of devaluation, shame, unworthiness, and guilt caused by applying the negative beliefs of society concerning a stigmatized attribute to the self [5,13].

Substance use stigma experiences are largely influenced by an individual’s interaction with their immediate physical, social, and cultural environments and the people around them [1,2,14,15]. Thus, it is valuable to adopt an ecological approach to examine the significant determinants at different levels of influence [16]. While similar approaches have been used in research concerning other groups, particularly persons who experience HIV stigma [17,18], there is a notable gap in understanding stigma in the context of substance use from a social ecological perspective.

We adapted the social ecological framework (SEF), which is frequently used for health promotion and intervention design [16], to hypothesize key constructs related to substance use stigma. The SEF is particularly suited for this research because it offers a comprehensive perspective that captures the influence of factors across multiple levels, including individual, interpersonal, contextual, community, and societal. This makes it a robust framework for interpreting health behaviors and developing interventions at the population level [16]. In addition, the SEF is a flexible framework that can be applied to focus on specific contexts, often referred to as settings [19,20]. Adapting from definitions in previous research, we defined *behavior settings* to be physical and conceptual settings where substance use stigma takes place [19,21]. We identified 5 behavior settings at the contextual level of influence in the SEF—home, school, work, leisure, and health care—that are potentially insightful for examining stigma manifestations and 5 *actors*—family, partners, friends, coworkers, and health care providers—who may play a role in these scenarios.

Using the ecological features, we aimed to identify characteristic ways in which stigma manifests, or *stigma phenotypes*, from social media data to better understand experiences of stigma in diverse social ecological contexts. We conceptualized a *stigma phenotype* as a distinct combination of features along 4 key dimensions: stigma mechanism, substance, setting, and actor. We sought to identify the patterns of co-occurrence of *stigma mechanisms* (internalized, anticipated, and/or enacted) with respect to different *substances* (alcohol, cannabis, and/or opioids) within diverse *settings* (home, work, school, leisure, and/or health care) and involving different *actors* (family, partners, and/or friends). The identification of stigma phenotypes can, in turn, provide valuable insights that inform the design of customized interventions tailored to different target groups.

There is a significant gap in the literature regarding the use of extensive, naturally occurring data to analyze experiences of substance use stigma. Most of the extant literature on this topic has used survey or interview methods, typically involving small to moderate sample sizes [9]. Social media enables the collection of extensive, naturally occurring data, which is not easily achievable in laboratory settings [22]. Reddit has been frequently used in substance use research as it provides a rich body of pseudoanonymous, user-generated content and features subreddits, or discussion forums, dedicated to topics related to substance use [23-26]. Given the volume of data, we leveraged natural language processing (NLP) and machine learning (ML) methods to derive the defining features of stigma phenotypes.

### Objectives

The purpose of this study was to examine stigma phenotypes in social media data to ensure a comprehensive and nuanced understanding of how substance use stigma operates within and across different ecological contexts. Cluster analysis is an unsupervised ML method that can reveal hidden patterns within a dataset and groups data points into clusters based on identified patterns [27]. Due to its ability to identify natural groupings within datasets [27], cluster analysis methods have been frequently used in biomedical research to extract disease phenotypes [28-30]. We performed cluster analysis to extract stigma phenotypes delineated by stigma mechanisms, substances, and ecological factors (behavior settings and actors) from an extensive social media dataset. In addition, we sought to investigate the differences between stigma phenotypes with respect to more granular ecological factors. We propose the following research questions (RQs):

1. What stigma phenotypes (defined by stigma mechanism, substance, setting, and actors) are reflected in social media data?
2. What additional ecological factors might influence these stigma phenotypes?

The identification of stigma phenotypes offers valuable insights in guiding the design and development of targeted interventions that address the specific needs and challenges faced by different groups experiencing substance use stigma. By leveraging these phenotypes, targeted interventions can acknowledge the heterogeneity in stigmatizing experiences and deliver support that is contextually relevant to individuals’ lived experiences.

## Methods

### Data Collection

**Figure 1:**
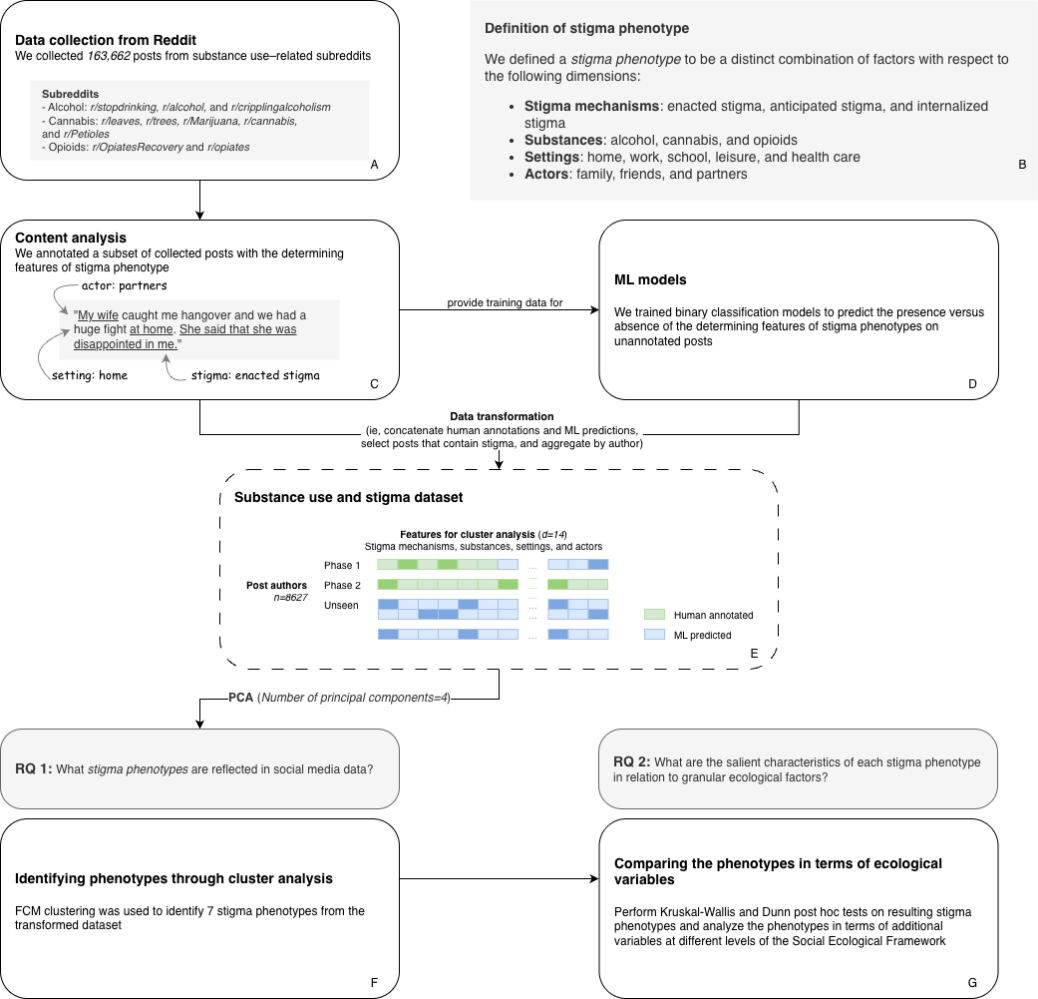
Study flowchart. FCM: fuzzy c-means; ML: machine learning; PCA: principal component analysis; RQ: research question.

We collected a total of 163,662 Reddit posts published between January 1, 2013, and December 31, 2019, from 10 subreddits (Figure 1, box A) using the Pushshift application programming interface [31]. Each subreddit focuses on 1 of our 3 substances of interest. Some subreddits (eg, r/OpiatesRecovery, r/leaves, and r/stopdrinking) are recovery focused, whereas the others (eg, r/opiates, r/cannabis, and r/cripplingalcoholism) are not. We only collected thread-initiating posts as they tend to contain richer information than their replies [32]. More information about the data collection process is discussed in our prior work [33].

### Feature Selection

A total of 14 features (ie, factors) were included in the cluster analysis (Figure 1, box B). There were 4 groups of features: substances (alcohol, cannabis, and/or opioids), stigma mechanisms (internalized stigma, anticipated stigma, and/or enacted stigma), settings (home, school, work, health care, and/or leisure), and actors (family, partners, and/or friends). The settings refer to both physical and conceptual settings; for example, *home* refers to both the presence or absence of the physical space and the idea of home. As such, if an individual mentioned housing insecurity in the passage (eg, being forced to leave their home, which could be important in the context of substance use [34]), this incident would be captured in the data. Specifically, *leisure* was defined as recreational situations in which a social element is present or implied. Each feature could take 1 of 2 values: 0 (absent) or 1 (present).

### Feature Engineering

**Figure 2:**
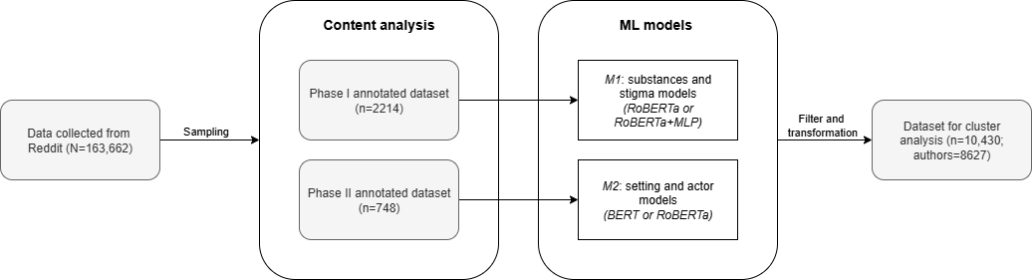
Feature engineering flowchart. BERT: bidirectional encoder representations from transformers; MLP: multilayer perceptron.

We used NLP methods to develop ML classifiers [35,36] that predicted the presence of each variable. We then used the classifiers to generate predictions on unseen data to be used for subsequent analyses. The feature engineering pipeline consisted of 2 main parts: content analysis (Figure 1, box C) and model development (Figure 1, box D). We conducted 2 phases of content analysis that corresponded to 2 sets of ML models, each serving as a foundation to derive different features necessary for the subsequent cluster analysis (Figure 2).

The phase 1 annotated dataset (n=2214 posts) was used to train models that predict the presence versus absence of the substances and stigma mechanisms [33], referred to as M1 models. The posts in this dataset were sampled using the keyword sampling approach described by Chen et al [12] to select posts that likely contained stigma. Posts were annotated with substances and stigma mechanisms. Further details on the operationalization of the stigma mechanisms are provided in Multimedia Appendix 1.

All the M1 models involved fine-tuning a pretrained RoBERTa model [35]. As stigma-related information is often not explicitly stated in text and requires interpretation beyond surface-level content, we incorporated an additional multilayer perceptron into the M1 models. The multilayer perceptron leveraged a variety of features, including term frequency–inverse document frequency weighted n-grams, the National Research Council Canada Emotion Intensity Lexicon [37], the WordNet-Affect Lexicon [38], Linguistic Inquiry and Word Count features [39], and other handcrafted features [33]. We report the *F*_1_-score as the primary evaluation metric for all models because it balances precision and recall, which is appropriate given that we do not have a strong preference between the 2 metrics. M1 substance models achieved an average *F*_1_-score of 0.96 (SD 0.01), whereas M1 stigma models achieved an average *F*_1_-score of 0.80 (SD 0.06). Further details on M1 model design, development, and evaluation can be found in our prior work [33].

In phase 2, we sought to identify a wider range of phenomena, including ecological features such as settings and actors. The phase 2 annotated dataset (n=748 posts) included 496 posts from the phase 1 annotated dataset and 252 posts added through quota sampling involving a balanced representation of each substance and stigma type to enrich the representation of stigma within the dataset. This dataset was used to develop classifiers that predicted settings and actors, referred to as M2 models. M2 models used only transformer-based architectures—either bidirectional encoder representations from transformers (BERT) or RoBERTa. To address challenges such as limited training data size and imbalanced data, we implemented the BERTprepend [40] data augmentation method on the training set. We then fine-tuned pretrained BERT and RoBERTa models [41,42] on the augmented dataset and selected the better-performing model for each feature. BERT achieved better performance for home, leisure, work, health care, and friends, and RoBERTa was better at predicting school, family, and partners. The M2 BERT and RoBERTa models achieved an average *F*_1_-score of 0.83 (SD 0.03) and 0.81 (SD 0.16), respectively, across the held-out folds of 5-fold cross-validation. Table 1 presents a summary of the model architectures, predicted features, and model performances.

**Table 1:** Overview of machine learning–based binary classification models used in feature engineering, showing model architectures, target variables, training and evaluation datasets, and model performance measured using the average F1-score.

Model and architecture		Target variables	Dataset	*F*_1_-score, mean (SD)
**M1^a^ models**				
	RoBERTa+MLP^b^	Stigma mechanisms: enacted stigma, anticipated stigma, and internalized stigma	Phase 1 annotated dataset	0.80 (0.06)
	RoBERTa	Substances: alcohol, cannabis, and opioids	Phase 1 annotated dataset	0.96 (0.01)
**M2^c^ models**				
	BERT^d^	Settings: home, leisure, work, and health care; actors: friends	Phase 2 annotated dataset	0.83 (0.03)
	RoBERTa	Settings: school; actors: family and partners	Phase 2 annotated dataset	0.81 (0.16)

^a^M1: ML models that predict stigma mechanisms and substances.

^b^MLP: multilayer perceptron.

^c^M2: ML models that predict settings and actors.

^d^BERT: bidirectional encoder representations from transformers.

### Identifying Phenotypes Through Cluster Analysis

As most collected posts did not contain stigma, we filtered the cluster analysis dataset to retain only stigma-positive posts, ensuring that the analyses were exclusively focused on stigma-related content. Once the features were derived, we restructured the dataset from post level to author level, aggregating the values of each feature across all posts written by the same person (Figure 1, box E). This procedure was performed to focus on understanding the stigma-related experiences of each individual on a holistic level.

To address RQ 1 (Figure 1, box F), we used the fuzzy c-means (FCM) clustering algorithm to derive stigma phenotypes from the data [43]. As we explored the appropriate clustering techniques, we evaluated a variety of methods, including but not limited to centroid-based, hierarchical clustering and fuzzy or soft clustering methods [44]. Unlike hard clustering methods that assign each case to a single cluster, FCM offers flexibility in handling ambiguous and complex data through partial cluster membership [45], making it particularly well suited for the analysis of substance use stigma, which often manifests in layered and nuanced ways.

With 14 features, we encountered the “curse of dimensionality” [46], meaning that an inverse relationship between meaningful differentiation between clusters and the number of features (or dimensions) was observed. To address this problem, we used principal component analysis (PCA) to condense the feature space down to 4 dimensions while preserving >50% of the cumulative explained variance in our data.

**Figure 3:**
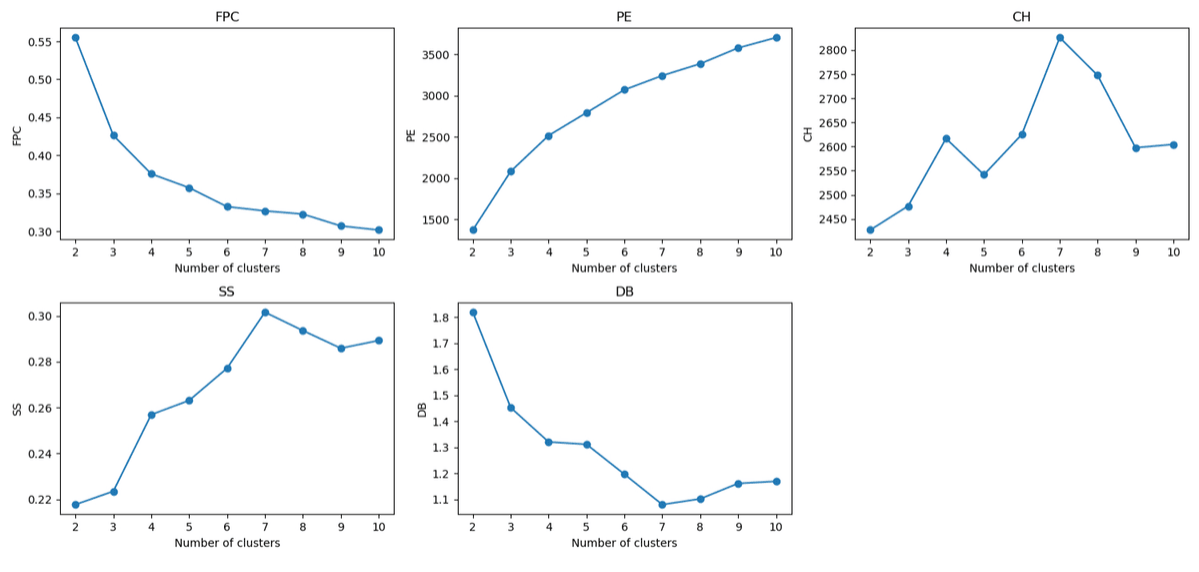
Validity indices for determining the optimal number of clusters c*. Five validity indices (fuzzy partition coefficient [FPC], partition entropy [PE], Calinski-Harabasz index [CH], silhouette score [SS], and Davies-Bouldin index [DB]) evaluated across 2 to 10 clusters to identify the optimal c* for deriving stigma phenotypes.

We used a range of validity indexes to determine the optimal number of clusters (c*) [47]—fuzzy partition coefficient, partition entropy, Calinski-Harabasz index, silhouette score, and Davies-Bouldin index (Figure 3). The fuzzy partition coefficient and partition entropy favored the smallest value (c*=2), which was not ideal for understanding complex and fuzzy stigma phenotypic patterns. In contrast, the Calinski-Harabasz index, silhouette score, and Davies-Bouldin index metrics, which measure clustering quality based on intercluster separation and intracluster compactness [48], were all in favor of c*=7 compared to other numbers. In summary, we used the FCM clustering algorithm to derive 7 stigma phenotypes from a PCA-transformed dataset with 4 principal components reduced from 14 raw features.

### Comparing the Phenotypes in Terms of Ecological Variables

In cluster analysis, it is common to include the dimensions that the researcher considers to be the defining characteristics, or the primary features of interest, as the variables to be clustered upon. Post hoc analyses are performed subsequently to better understand factors that may influence the defining characteristics [49].

**Figure 4:**
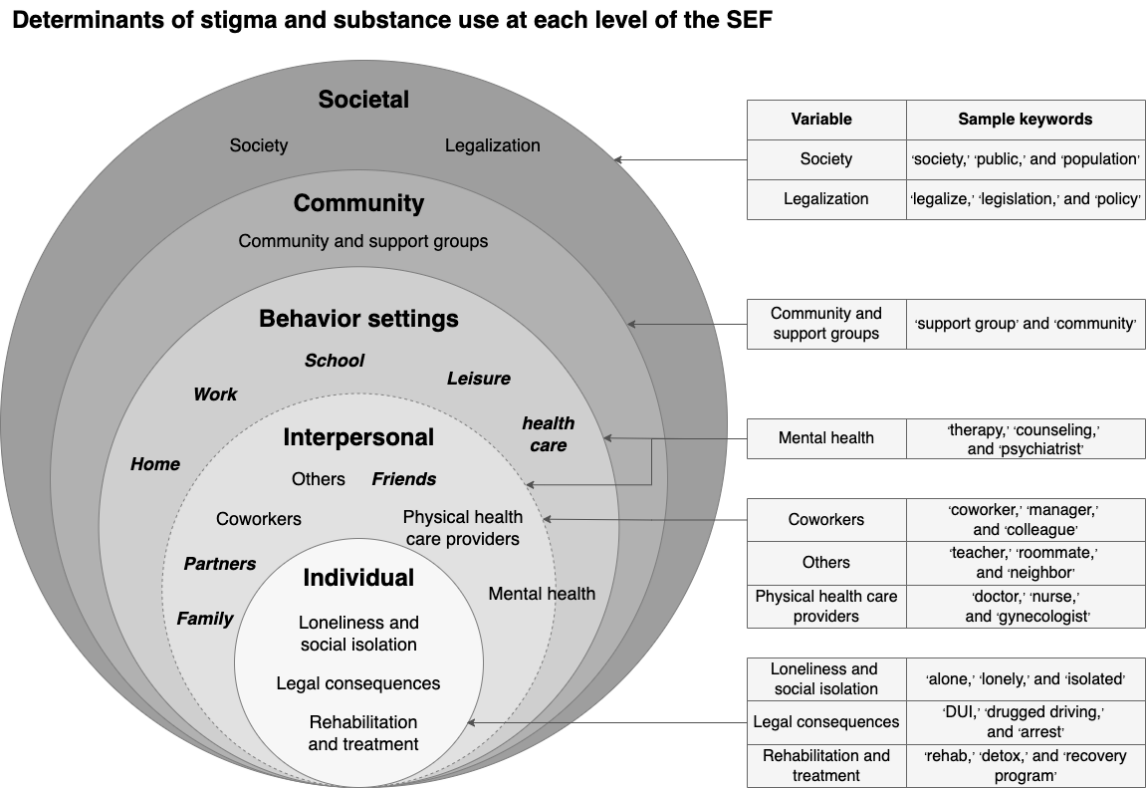
Social ecological framework (SEF) for determinants of substance use stigma at different levels of influence and sample keywords used for keyword-based statistical analysis.

In this study, the primary characteristics of interest were the settings and actors that might be involved in stigma-related experiences, and thus, these served as the focus of RQ 1. However, also consistent with an ecological view, human activity is influenced by a myriad of other factors that intersect at different levels. Thus, for RQ 2 (Figure 1, box G), we sought to identify additional factors that shape substance use stigma experiences at different levels of influence guided by the SEF [16,50] and compare their relative prominence with respect to the phenotypes (Figure 4).

We used a keyword-based approach to systematically capture ecological variables. The full list of keywords can be found in Multimedia Appendix 2. We developed keyword lists and then normalized keyword counts by calculating the percentage of sentences containing the keywords, which allowed for fair and meaningful comparisons across posts of varying lengths.

At the individual level, *loneliness and social isolation* were related to substance use and stigma [51-53]. Through content analysis, a systematic method of assigning codes to text segments [54], we observed that *legal consequences* and *rehabilitation and treatment* were frequent themes in the dataset. For example, individuals can be stigmatized for driving under the influence of a substance, and people may choose to engage in rehabilitation, treatment, or therapy; capturing these could facilitate a more comprehensive view of the challenges that those experiencing substance use stigma might face.

At the interpersonal level, the actors included *family*, *partners*, and *friends*, which were prominent actors in the dataset and were included in cluster analysis. Interactions with other actors such as *coworkers*, *health care providers*, and *others* (eg, servers, roommates, and barbers) can also play critical a role in the experiences of a stigmatized individual and, thus, were included in the post hoc analyses.

Behavior settings are physical and conceptual settings in which stigma and substance use experiences take place [19]. *Home*, *work*, *school*, *leisure*, and *health care* are prominent settings that were included in the cluster analysis. It is important to note that the interpersonal variables and behavior settings are often interconnected but do not completely overlap. For example, one can experience stigma at home, but the stigma is not necessarily enacted by family or partners. *Mental health care* sits at the intersection of the interpersonal level and behavior settings as it covers both the mental health care setting and mental health care providers.

At the community level, we included the variable *community and support groups*, which covers both online and offline communities and support groups such as Alcoholics Anonymous. This is an important variable as communities (primarily online communities, referring to subreddits in this dataset) and support groups can help individuals succeed at substance use recovery [55,56]. At the societal level, *legalization* of substances is an important factor to consider as individuals who use illicit substances often experience higher levels of stigma [57]; *society* itself is also an actor.

We used statistical tests to examine the differences among the 7 stigma phenotypes regarding these granular variables. As interim multinomial logistic regression results showed minimal to no interaction effects, we independently analyzed differences between the clusters on the granular ecological variables using the nonparametric Kruskal-Wallis test. To account for multiple comparisons, we performed the Dunn post hoc test with Bonferroni adjustment. These methods are ideal for comparing the phenotypes on nonnormally distributed keyword-based variables [58]. The full set of test statistics can be found in Multimedia Appendix 3.

### Ethical Considerations

In this paper, we use synthetic or paraphrased quotes derived from original data to provide examples of key themes in the data. We use synthetic quotes to protect author privacy as direct quotes from online sources could be traced back to their original posts [59]. As we developed the synthetic quotes, we worked to ensure that the quotes were representative of the raw data and accurately reflected the language, meaning, and connotation of the original content [60]. This study received ethics approval from the University of Washington Human Subjects Division (STUDY00015737) and the University of Melbourne Human Research Ethics Committee (2023-25512-48127).

## Results

### Sample Statistics

Our sample comprised 10,430 stigma-related posts representing 8627 authors. Table 2 shows the descriptive statistics of the dataset at both the author and post levels. The numbers reported in the text pertain to the author level.

**Table 2:** Sample characteristics regarding feature presence at the author and post levels based on predictions from the machine learning models.

Feature			Author level (n=8627), n (%)		Post level (n=10,430), n (%)
**Substances**					
	Alcohol	4571 (52.98)		5510 (52.83)	
	Cannabis	3156 (36.58)		3680 (35.28)	
	Opioids	1288 (14.93)		1552 (14.88)	
**Stigma mechanisms**					
	Enacted stigma	3390 (39.3)		3832 (36.74)	
	Anticipated stigma	2767 (32.07)		3031 (29.06)	
	Internalized stigma	4823 (55.91)		5560 (53.31)	
**Settings**					
	Home	3590 (41.61)		4003 (38.38)	
	Work	2753 (31.91)		3038 (29.13)	
	School	1358 (15.74)		1437 (13.78)	
	Leisure	2784 (32.27)		3035 (29.1)	
	Health care	2016 (23.37)		2202 (21.11)	
**Actors**					
	Family	3616 (41.91)		4034 (38.68)	
	Partners	2720 (31.53)		2991 (28.68)	
	Friends	3013 (34.93)		3244 (31.1)	

Among the sample, the proportion of authors mentioning each substance varied, with 52.98% (4571/8627) mentioning alcohol, 36.58% (3156/8627) mentioning cannabis, and 14.93% (1288/8627) mentioning opioids. Most authors (7690/8627, 89.1%) indicated use of a single substance within the scope of the posts captured in this dataset.

Internalized stigma (4823/8627, 55.91%) was the most prominent stigma mechanism, more common than enacted stigma (3390/8627, 39.3%) and anticipated stigma (2767/8627, 32.07%). Among the ecological variables, home (3590/8627, 41.61%) and family (3616/8627, 41.91%) were more prominent, whereas school (1358/8627, 15.74%) and health care (2016/8627, 23.37%) were less common.

### The Stigma Phenotypes

#### Overview

We identified 7 distinct stigma phenotypes from this dataset. Table 3 summarizes the average post length of each phenotype.

**Table 3:** Post length statistics by stigma phenotype.

Phenotype ID	Authors (n=8627), n (%)	Number of words, mean (SD)	Number of words, median (IQR)
0	1217 (14.1)	309 (322)	223 (235)
1	1418 (16.44)	381 (277)	310 (246)
2	1363 (15.8)	177 (170)	137 (131)
3	1037 (12.02)	233 (328)	152 (223)
4	1153 (13.37)	433 (351)	344 (307)
5	1287 (14.92)	259 (292)	199 (182)
6	1152 (13.35)	200 (185)	154 (164)

**Figure 5:**
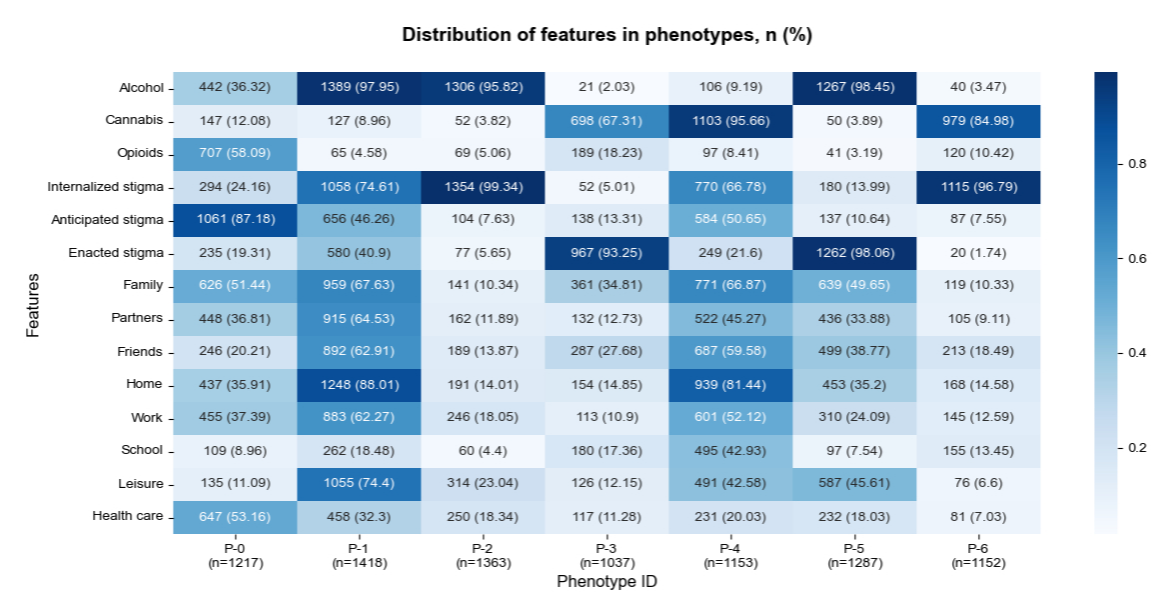
Distribution of substances, stigma mechanisms, actors, and settings across 7 identified stigma phenotypes. The heatmap is colored based on the percentage of feature presence in each phenotype, with darker colors indicating stronger presence.

Figure 5 illustrates the distribution of features in each stigma phenotype, with darker cells indicating higher presence and lighter cells representing lower presence. Even though PCA was used to reduce dimensionality, the interpretability of transformed components is conveyed through their alignment with original dimensions. Thus, the reporting of statistics in the characterization of stigma phenotypes was guided by the input features that were used to derive the principal components. The stigma mechanisms and substances were the primary defining characteristics of the clusters, and the setting and actor features contributed to the differentiation between clusters in a more nuanced manner. The phenotypes were as follows:

1. *opioids/alcohol–anticipated stigma–health care* (P-0)
2. *alcohol–mixed stigma–more settings and actors* (P-1)
3. *alcohol–internalized stigma–few settings and actors* (P-2)
4. *cannabis/opioids–enacted stigma–few settings and actors* (P-3)
5. *cannabis–mixed stigma–more settings and actors* (P-4)
6. *alcohol–enacted stigma–few settings and actors* (P-5)
7. *cannabis–internalized stigma–few settings and actors*

The 7 phenotypes can be broadly categorized into 2 groups distinguished by shared presence or absence of certain features: those characterized by a single prominent stigma mechanism (P-0, P-2, P-3, P-5, and P-6) and those displaying moderate to high prominence of multiple stigma mechanisms (P-1 and P-4). Among the single-stigma group, there was a distinct separation of the 3 stigma types, with P-2 and P-6 characterized by internalized stigma, P-0 characterized by anticipated stigma, and P-3 and P-5 characterized by enacted stigma.

#### Single-Stigma Phenotypes

Phenotype 2 (P-2; 1363/8627, 15.8% of the authors) and phenotype 6 (P-6; 1152/8627, 13.35% of the authors) were characterized by a high presence of internalized stigma and low presence of anticipated and enacted stigma. The main difference between P-2 and P-6 was the predominant substance—P-2 authors primarily used alcohol (1306/1363, 95.82%), whereas P-6 authors mainly used cannabis (979/1152, 84.98%), with a small proportion using opioids (120/1152, 10.42%). The posts were relatively short (Table 3), and settings and actors were rarely mentioned in both phenotypes. The narratives in P-2 and P-6 were usually centered on the posters’ internal feelings and thoughts, without providing much detail about the context:

I am embarrassed by my drunk actions. I feel like I’m not capable of anything.

P-2; internalized stigma; alcohol

There were 2 phenotypes with enacted stigma as the distinguishing characteristic (P-3, 1037/8627, 12.02% of the authors and P-5, 1287/8627, 14.92% of the authors). P-5 was characterized by alcohol use (1267/1287, 98.45%), whereas P-3 was mainly associated with cannabis use (698/1037, 67.31%), with some authors (189/1037, 18.23%) reporting opioid use. P-3 and P-5 also differed in terms of the involvement of ecological features. Settings and actors such as family, friends, partners, home, and leisure were common themes in P-5; however, the presence of settings and actors was lower in P-3, except for school. In both enacted stigma phenotypes, it was common for the narratives to center on specific situations, with family and friends being frequently mentioned:

My wife caught me hung over and we had a huge fight at home. She said that she was disappointed in me, and it really hurts.

P-5; enacted stigma; alcohol

Phenotype 0 (P-0; 1217/8627, 14.11% of the authors) was the only phenotype in which anticipated stigma was the leading stigma mechanism (1051/1217, 86.36%). Internalized stigma and enacted stigma were also experienced by some individuals but to a lesser extent. The substances used by P-0 authors varied, with 58.09% (707/1217) reporting opioid use, 36.32% (442/1217) reporting alcohol use, and 12.08% (147/1217) reporting cannabis use. Most settings and actors were moderately common, except for school and leisure.

Notably, P-0 was the only phenotype in which health care was the most prominent setting (647/1217, 53.16%). In many cases, anticipated stigma and health care coexisted without being directly related; however, there were scenarios in which the connections between the 2 features were evident. Some individuals felt the need to seek help from therapists because they hid their substance use problems from people around them, and some others anticipated stigma from health care providers:

I need to talk to a therapist about my situation because there’s no one else I can talk to. My wife and my family can’t find out that I’m using again.

P-0; anticipated stigma; health care

How do I get my Dr to prescribe more pain killers without me appearing like a drug seeker?

P-0; anticipated stigma; health care

#### Mixed-Stigma Phenotypes

There were 2 phenotypes in which authors experienced multiple stigma mechanisms. Phenotype 1 (P-1; 1418/8627, 16.44% of the authors) was characterized by alcohol use (1389/1418, 97.95%). In P-1, a total of 74.61% (1058/1418) of the authors mentioned internalized stigma, 46.26% (656/1418) mentioned anticipated stigma, and 40.9% (580/1418) mentioned enacted stigma. Phenotype 4 (P-4; 1153/8627, 13.37%) was characterized by cannabis use (1103/1153, 95.66%). In total, 66.78% (770/1153) of P-4 authors discussed internalized stigma, 50.65% (584/1153) mentioned anticipated stigma, and 21.6% (249/1153) discussed enacted stigma. Narratives in P-1 and P-4 were lengthier (P-1: mean 381, SD 277 words; P-4: mean 433, SD 351 words) and more likely to involve settings and actors. The interconnection between different stigma mechanisms was evident in P-1 and P-4 posts. For example, many authors discussed feelings of internalized stigma and past experiences of enacted stigma:

How do you all deal with the constant feeling of shame? I keep replaying what happened the other day: I got drunk, blacked out, and ended up vomiting in my friend’s car. I could tell my friend was judging me, and I totally deserve it. I feel so guilty and ashamed, and I can’t stop thinking about it. I really want to quit drinking.

P-1; alcohol; enacted stigma; internalized stigma; friends

In addition, there were notable differences between phenotypes characterized by different substances. In the internalized stigma–only, enacted stigma–only, and mixed-stigma groups, the school setting was consistently more prominent in the cannabis phenotypes (P-6, P-3, and P-4) compared to the alcohol phenotypes (P-2, P-5, and P-1). Although not many authors explicitly discussed incidents occurring at school, many mentioned that they began smoking cannabis while they were students. Conversely, the leisure setting was consistently more prominent in the alcohol phenotypes than in their cannabis and opioid counterparts. Authors frequently referred to leisure settings to provide context for their stories, but there were also instances in which they discussed stigma-related challenges encountered in leisure contexts:

I’m excited to attend my friend’s wedding, but I’m worried about what I should say when they offer me drinks. None of them knows that I am an alcoholic and have been trying to stay sober.

P-1; alcohol; anticipated stigma; leisure

### Comparing the Phenotypes in Terms of Ecological Variables

We conducted Kruskal-Wallis and Dunn post hoc tests with Bonferroni correction to compare and analyze the phenotypes with respect to granular ecological variables. In this section, we focus on the most meaningful comparisons, which are shown in Table 4. The unadjusted and adjusted *P* values for the comparisons between each combination of phenotypes can be found in Multimedia Appendix 3.

**Table 4:** Notable statistically significant pairwise comparisons from Kruskal-Wallis and Dunn post hoc tests with Bonferroni correction (adjusted P<.05)^a^.

Level and variable		Notable comparisons
**Individual**		
	Loneliness and social isolation	P-1 and P-4>other phenotypes^b^
	Rehabilitation and treatment	P-0>other phenotypes P-1>other phenotypes except for P-0 P-5>P-3, P-4, and P-6
	Legal consequences	P-3>other phenotypes
**Interpersonal**		
	Coworkers	P-1<other phenotypes
	Physical health care providers	P-0>other phenotypes
	Others	P-1>P-0, P-2, P-3, P-5, and P-6 P-4>P-0, P-2, P-3, and P-6 P-5>P-0, P-2, and P-6
**Behavior settings**		
	Mental health care	P-0, P-1, and P-4>other phenotypes
**Community**		
	Community and support groups	P-1>other phenotypes
**Society**		
	Society	P-3>P-0, P-2, P-5, and P-6 P-1>P-2, P-5, and P-6
	Legalization	P-3>other phenotypes

^a^P-0 to P-6 denote the stigma phenotypes derived from cluster analysis. See text above for pattern summaries.

bOther phenotypes refers to the set of phenotypes not explicitly listed on the left side of the comparison. For example, in P-1 and P-4>other phenotypes, other phenotypes refers to P-0, P-2, P-3, P-5, and P-6.

In Table 4, we observe that, at the individual level, the mixed-stigma phenotypes (P-1 and P-4) exhibited significantly higher levels of *loneliness and social isolation* than other phenotypes (Table S1 in Multimedia Appendix 3). The notation *P-1 and P-4>other phenotypes* indicates that phenotype 1 was significantly greater with respect to this variable when individually compared to the other phenotypes; it does not imply that P-1 and P-4 are significantly different from each other. *Rehabilitation and treatment* was highest in P-0—characterized by opioid and alcohol use, anticipated stigma, and health care—followed by 2 other alcohol phenotypes, P-1 and P-5 (Table S2 in Multimedia Appendix 3). *Legal consequences* were notably higher in P-3 (Table S3 in Multimedia Appendix 3), which is characterized by cannabis and opioid use, enacted stigma, and relatively low presence of the settings and actors studied:

I got pulled over a couple days ago. The cops thought I was high, but I was completely sober. Then they searched me without even asking for my permission.

P-3; legal consequences

At the interpersonal level, *coworkers* was significantly higher in P-1 (alcohol; mixed stigma) than in other phenotypes (Table S4 in Multimedia Appendix 3). *Physical health care providers* were mentioned more in P-0, where health care was the most prominent setting (Table S5 in Multimedia Appendix 3). *Others* were most frequently mentioned in P-1, followed by P-4 (cannabis; mixed stigma) and P-5 (alcohol; enacted stigma; Table S6 in Multimedia Appendix 3). P-1, P-4, and P-5 were phenotypes with higher presence of actors in general. The behavior setting *mental health care* was more prominent in P-0, P-1, and P-4 (Table S7 in Multimedia Appendix 3).

At the community level, P-1 authors mentioned *community and support groups* more frequently than authors in other phenotypes (Table S8 in Multimedia Appendix 3). There was a strong sense of (online) community among P-1 authors:

34 days sober. I could really use some words of encouragement to support me through the process, and I will stay active in this thread for accountability. Thanks everyone.

P-1; alcohol; community and support groups

Finally, at the societal level, *society* was mentioned more by P-3 and P-1 authors compared to authors in some but not all other phenotypes (P-2, P-5, and P-6; Table S9 in Multimedia Appendix 3). P-3 authors, characterized by opioid or cannabis use, discussed *legalization* much more than those in other groups (Table S10 in Multimedia Appendix 3).

## Discussion

### Principal Findings

Using the FCM clustering algorithm, we derived 7 stigma phenotypes based on posts containing stigma related to 3 substances. Stigma mechanisms and substances were the key determinants of the phenotypes, and settings and actors influenced the differentiation between clusters in a more subtle way. The 7 stigma phenotypes can be categorized into 4 groups: internalized stigma–only group (P-2 and P-6), anticipated stigma group (P-0), enacted stigma–only group (P-3 and P-5), and mixed-stigma group (P-1 and P-4). This distinction highlights significant differences in the manifestations of the stigma mechanisms individually and in concert.

The narratives in the internalized stigma–only phenotypes were characterized by a focus on internal feelings, with few references to contextual factors. In contrast, the narratives in the enacted and anticipated stigma phenotypes usually involved more settings and actors, which exemplifies the inherently interpersonal nature of these 2 stigma mechanisms [11].

We observed a complex connection among anticipated stigma, health care, and opioid use in the anticipated stigma phenotype (P-0), which was the only phenotype where anticipated stigma, opioid use, and health care were significantly more common than other stigma types, substances, and settings, respectively. Health care providers often exhibit negative attitudes toward patients with substance use disorders [61]; consequently, individuals with substance use problems frequently experience anticipated stigma in health care settings, reducing their willingness to seek help from health care professionals [62,63]. This pattern is particularly pronounced among opioid users, exemplified by behaviors such as attempting to hide their substance use history and avoiding health care interactions [4]. Moreover, the high prevalence of the mental health care setting in this phenotype is likely related to anticipated stigma—individuals often seek or consider seeking help from mental health care providers because they find it difficult to discuss their issues with people around them.

In the mixed-stigma phenotypes (P-1 and P-4), the connections between stigma mechanisms were evident. We observed that past experiences of enacted stigma can lead to or reinforce anticipated and internalized stigma [64]. This is in line with the progressive model of self-stigma [65], which posits 4 stages that eventually lead to diminished self-esteem. This first stage is being aware of the stereotypes, which can be caused by incidents of enacted stigma and may result in anticipated stigma. Individuals may then agree with the stereotypes and apply the stereotypes to themselves, which aligns with internalized stigma. Finally, the internalized stigma that individuals experience may lead to lower self-esteem.

In addition, significantly higher levels of loneliness and social isolation were observed in the mixed-stigma phenotypes. While the underlying mechanisms behind this phenomenon remain unclear, several possibilities can be suggested. In some cases, individuals may isolate themselves voluntarily to avoid social interactions and social stigma [66]. Moreover, loneliness and social isolation may also reinforce both anticipated and internalized stigma as individuals continually worry about how others perceive and react to them [66]. There was also a high presence of the mental health care setting in the mixed-stigma group. Given these results, interventions targeting individuals who experience multiple forms of stigma should address the individuals’ feelings of loneliness and social isolation and needs for therapy.

Moreover, substantial differences were evident among the substances studied. The leisure setting was more prominent in alcohol phenotypes than in their cannabis and opioid counterparts. Individuals recovering from alcohol use may feel social pressure to drink and experience stigma in leisure contexts as they attempt to hide their substance-related problems. Therefore, interventions for alcohol users could focus on stigma reduction in social and leisure contexts. The school setting was more prominent in cannabis phenotypes. Previous research has demonstrated that cannabis use is notably prevalent among middle and high school students [67,68], and exposure to cannabis use at school increases the likelihood of students’ own cannabis use, but the same pattern does not hold for alcohol [69]. Our finding further solidifies the necessity for targeted interventions designed for school settings [70].

Furthermore, there were distinctive ways in which specific phenotypes stood out. P-0, characterized by opioid and alcohol use, was the only cluster in which health care was the most prominent setting. It was common for P-0 authors to mention chronic pain conditions and the use of opioids for pain management [71]. In addition, there were also cases in which the authors engaged with physical health care providers due to consequences of opioid or alcohol use, such as overdose, severe hangovers, or driving under the influence accidents. These results suggest that interventions in the health care scope should address not only the consequences but also the underlying circumstances of substance use.

In comparison to all other phenotypes, the role of community and support groups was highly emphasized in P-1, which was characterized by alcohol and mixed-stigma mechanisms. P-1 individuals had a strong sense of (online) community—they sought social support from each other and provided support when they could. They also used the online community to hold themselves accountable. In addition, offline support groups such as Alcoholics Anonymous were often mentioned in P-1 narratives. Congruent with our own prior work, individuals expressed concerns and uncertainties about attending offline support groups, shared both positive and negative experiences and insights, encouraged others to join, or discussed why support groups did not work well for them [12]. These findings suggest that online communities such as Reddit can serve as valuable resources for individuals with substance use stigma problems. Offline support groups can also provide support and offer a structured environment for recovery, but they may not be suitable for everyone. While P-3 and P-5 were both characterized by enacted stigma, the involvement of actors and settings was notably lower in P-3 (cannabis and opioids) than in P-5 (alcohol). This discrepancy might be largely attributed to the prevalence of legal consequences, legalization, and society in P-3. Some individuals may express concerns regarding the legal consequences of their substance use behaviors. In other cases, legal consequences can manifest as a form of enacted stigma. For example, individuals may feel that they were unfairly stopped or searched due to their substance use history or appearing to be under the influence. At the societal level, the legalization of a substance can influence the public’s attitudes toward the normalization of its use [6]. Individuals in P-3 actively engaged in discussions about cannabis legalization, covering a range of topics. They expressed their opinions on government policies, shared troubles they faced due to the legal status of the substance they used, and discussed how drug laws disproportionately affect ethnic minority groups. These findings highlight the need for interventions to consider the impacts of legal consequences, substance legalization, society, and the stigma associated with substance use.

### Strengths and Limitations

This research has the following contributions and strengths. This study examined the manifestations of substance use–related stigma in a large-scale dataset. Unlike many studies that focus on a specific subgroup of individuals who either use a particular substance or experience a specific type of stigma [72,73], this research adopted a holistic approach to identify stigma phenotypes in a large dataset by including users of different substances and their experiences with different stigma mechanisms. Furthermore, even though NLP and ML techniques [74] and the SEF model [75,76] have been used separately in previous studies to explore health care–related topics, this research introduced a novel methodology that combined all 3. This combined approach ensured the scalability of the research by facilitating the understanding and analysis of a volume of data at a level of granularity that is difficult to achieve with qualitative and survey-based methods.

The limitations of this study are related to the nature of the dataset and sampling bias. The analysis was limited to Reddit data from 10 subreddits, which may not represent broader populations who experience substance use stigma, particularly those who are less engaged in social media. The percentage of authors who mentioned opioid use was significantly lower than the percentage of authors who mentioned the other 2 substances of interest, which could introduce bias in the clustering results. In addition, the results of this study largely depend on what the posters chose to disclose and how much detail they chose to include. In general, we found that phenotypes with longer average post lengths (P-0, P-1, and P-4) tended to have a higher presence of stigma, behavior settings, and actors. In phenotypes with shorter posts (P-2 and P-6), these factors were mostly absent. The relationship among post length, detail, and presence of features was subtle and complex. On the one hand, individuals who wrote longer, more detailed posts may have encountered problems in multiple aspects of their lives; thus, their discussions revolved around multiple settings, actors, and stigma mechanisms. On the other hand, they might simply feel a stronger urge of self-disclosure or have a detail-oriented writing style.

### Conclusions

In this study, we introduced a novel combination of NLP, ML, and statistical techniques to examine narratives containing stigma related to substance use from a social ecological perspective. We identified 7 phenotypes from large-scale social media data about substance use and stigma. These phenotypes revealed distinct patterns in terms of stigma mechanisms, substances used, settings and actors involved, and the role of ecological factors across different levels of our framework. The findings of this study provide a framework for understanding stigma as a multidimensional and context-dependent phenomenon. The valuable insights from this research could inform the design and development of interventions targeting different stigma contexts.

## Appendix

Multimedia Appendix 1 Annotation guide for stigma mechanisms, settings, and actors.

Multimedia Appendix 2 Keywords for social ecological variables.

Multimedia Appendix 3 Full results of the Kruskal-Wallis and Dunn post hoc tests.

## Abbreviations

BERT: bidirectional encoder representations from transformers
FCM: fuzzy c-means
ML: machine learning
NLP: natural language processing
PCA: principal component analysis
RQ: research question
SEF: social ecological framework

## Data Availability

The datasets generated or analyzed during this study are not publicly available due to privacy concerns as the datasets may contain sensitive information of social media post authors.
