# Supplementary material for: Identifying Stigma Phenotypes in Social Media Narratives of Substance Use: Observational Study
Source: J Med Internet Res. 2025 Nov 13;27:e68695. doi: 10.2196/68695 (PMC12661227; doi:10.2196/68695)
Supplement: Multimedia Appendix 1 [file jmir_v27i1e68695_app1.docx]

**Appendix 1. Annotation guide**

This appendix excerpts parts of our annotation guide, highlighting the parts most relevant to variables studied in this study: stigma mechanisms, settings, and actors.

**Stigma Mechanisms**

We are annotating probable occurrences of three different types of stigma: ***internalized, anticipated, and enacted* stigma.** Annotate as much of the text as needed to capture the instance of stigma. This could be part of a sentence, one sentence, or multiple sentences.

- **Enacted Stigma:** describes experiences of stereotyping, prejudice, and discrimination from others due to a stigmatized attribute.
  - Annotate situations in which people experience legal consequences due to substance use, such as receiving a DUI or being arrested.
  - Annotate this even if the causal attribution is not clear.
  - Do not annotate instances in which those who engage in substance use treat someone who has quit or is trying to, in a negative way.
  - Annotate situations in which stigma is expressed having to do with a substance that is used to quit the target substance in question (alcohol, cannabis, opioids). An example would be when a person criticizes the use of suboxone for quitting.
  - Annotate instances in which actual substance use is not mentioned (in other words, there is no substance annotation), but someone mentions enacted stigma relating to persons who uses substances more generally.
  - Take what the person says as at face value (accept what they perceive as reality, as opposed to trying to assess whether things are really as they say they are).
- **Anticipated Stigma:** The expectation that one might experience stereotyping, prejudice and discrimination from others due to a stigmatized attribute.
  - This would include: perceptions of society towards substance use, situations in which someone is hiding their habit, being secretive, deceiving others or lying about their habit, and stealing.
  - If a person says that they think that negative consequences would occur due to their substance use being found out, it could be considered anticipated stigma.
  - Annotate this even if the causal attribution is not clear.
  - Annotate instances in which someone is surprised that they were not treated badly due to their substance use, or instances in which someone anticipates that they will be treated with prejudice, even if that turns out to not be the case (e.g., a child expects that the parent will turn them out of the house, but the parent says that they understand and they will support them through their situation).
- **Internalized Stigma:** Individuals internalize negative stereotypes about the stigmatized group that they belong to and apply these to themselves, which can and often does affect their sense of self-worth (Kulesza et al., 2017).
  - This may involve self-incrimination in relation to substance use.
  - This may also be manifest as hopelessness and/or weakness (however, hopelessness and/or weakness on their own, is not enough to constitute internalized stigma).
  - We might consider a concept such as “hopelessness” carrying more weight if it is in the title. (For example, if hopelessness comes up in the title, we can annotate it as an indication of self-stigma due to its being in a substance use-related discussion forum.)
  - We assign “internalized stigma” when a person talks about feeling embarrassed, shameful, and worthless within. Those emotions especially when it is coupled with self-incriminating thoughts makes a case of “internalized stigma”.
  - Internalized stigma involves acceptance of the pejorative (negative attitudes, views, assumptions related to substance use)

**Do annotate:**

- Examples in which the poster is not the main actor involved in the stigma.

**Do not annotate:**

- Fictional stories or articles (identify stories of stigma that are actually true).
- Dreams.
- Predictions or hypothetical situations.
- Do not annotate stigma due to reasons other than substance use, unless they are mixed with substance use stigma. For example: do not annotate the expression of depression on its own, disconnected to feeling badly about one’s use of substances.
- Do not annotate stigma related to substances that we are not studying (e.g., LSD).

**Other notes:**

- Recognizing that you have a problem is not necessarily indicative of stigma (there is a difference between helpful self-reflection and self-stigmatization).
- Distinguish between stigma and substance use. Falling back into substance use is not an example of internalized stigma.
- When we see examples of stigma in the past, code them as stigma, except when the person says they no longer experience it. For example, if the person says they no longer feel shame or they no longer feel worthless, then do not code it as internalized stigma.
- “Loser”, can generally be annotated with “labeling” and “internalized stigma”.
- If a person says that the substance makes them lazy or results in negative consequences (such as getting into accidents), it is not necessarily indicative of stigma. We will annotate it as stigma if the passage seems to convey a logic where the person seems to feel that people who use that substance are lazy, and since they themselves use the substance, then they are lazy.
- Humiliation can be internal or external. When you encounter an instance of humiliation, think about whether the person is feeling humiliated (likely internalized stigma), or whether someone said something to them in response to something that they did (likely enacted stigma).
- Drug testing can be coded as enacted stigma and the anticipation of failing a drug test can be coded as anticipated stigma. We consider drug testing as an example of institutional stigma even when it is not clear whether a positive drug test result will result in negative consequences for a participant.

**Settings**

These may include school, work, home, healthcare spaces, and social events, recovery groups, social media, public spaces, retail environments.

- **Home:** the existence of a home, situations and factors relating to “*home life*”, home issues that arise because of substance use, etc. For instance, the “home” can be applied when the instance took place at home, when a person talks about using substances at home (without necessarily anything happening at home), or when a person talks about difficulties finding a residence (as it relates to home). This includes a temporary home.
- **Work:** mentions of a job, career, or place of work; situations and factors relating to “*work life*”, work issues related to substance use, etc.
- **School:** mentions of school, academics, homework, studies; situations relating to “*school life*” or academics, issues relating to school/academics because of substance use, etc.
  - The “School” code should be applied when it’s related to something happening in the context of school.  This might be when the person in the story is a student, a parent of a child is in school, among other situations.
  - Even when the person mentions something related to school just to specify when the incident had happened (e.g., when I was in high school), the “School” code still applies as it can contribute to the context the person was situated in.
  - If the person who is using substances happens to work at/be employed at a school, but the school context isn’t relevant to the story, the school code can be omitted.
- **Leisure:** recreational situations and environments that likely contain a social element or implied social element, such as parties, social events and gatherings, nightclubs and/or bars, etc. Individual leisure activities (such as playing video games, watching TV/Netflix, etc.) should not be coded for as “Leisure”
- **Healthcare:** situations and circumstances related to healthcare; mentions of medical settings (clinics, hospitals, ER, pharmacies, etc.), medical practices and treatments, telehealth, therapy, etc. Instances of rehabilitation and treatment can also be labeled as healthcare. Detox and cognitive behavioral therapy are not considered healthcare without sufficient context information suggesting that these practices took place in a healthcare setting.

**Actors**

These may include friends, family, parents, healthcare providers, children, partner, personal network (social circle), co-worker including manager or owner (note power dynamic), relative, hookup (connection), etc.

- **Code this whether or not the actor is playing a role in the stigma-related experience** (meaning, if friends are mentioned even if they are not the stigmatizer or the stigmatized, code it).
- Code this whether or not the actor is explicitly mentioned when you can assume the presence of certain actors in certain settings (meaning, if someone mentions going to a pharmacy without mentioning a pharmacist, still code ‘healthcare provider’; if someone mentions losing a reputation at work without mentioning coworkers, still code ‘coworkers’).
- Code this whether or not the actor is physically present in the setting. For instance, when someone mentions family history in substance use, we consider family as a relevant actor in the person’s experience, hence the code ‘family’.
- **Family:** parents, siblings, children, extended family and relatives (including step-family), etc.
  - For spouses, use the Partners code instead
- **Friends**
  - Roommates can be considered friends only if there is enough context in the post to describe a friendship or friendly relationship.
  - Friends made at workplace can be considered both friends and coworkers
- **Partners:** present and/or past spouses, significant others, ex-partners, etc, including a person they’ve gone on a few dates with (because they have a potential to be developed into a partnership).
- **Coworkers**
- **Healthcare providers:** doctors, nurses, therapists, counselors, etc.

**Reference**

Kulesza, M., Watkins, K. E., Ober, A. J., Osilla, K. C., & Ewing, B. (2017). Internalized stigma as an independent risk factor for substance use problems among primary care patients: Rationale and preliminary support. *Drug and Alcohol Dependence*, *180*, 52–55. https://doi.org/10.1016/j.drugalcdep.2017.08.002
