# Supplementary material for: Identifying Stigma Phenotypes in Social Media Narratives of Substance Use: Observational Study
Source: J Med Internet Res. 2025 Nov 13;27:e68695. doi: 10.2196/68695 (PMC12661227; doi:10.2196/68695)
Supplement: Multimedia Appendix 2 [file jmir_v27i1e68695_app2.docx]

**Appendix 2. Keywords for Social Ecological Variables**

| Variable | Keywords |
| --- | --- |
| Loneliness and social isolation | lonely, loneliness, social isolation, isolated, isolation, alone, solitude, unwanted, unloved, excluded, unappreciated, don’t go out, don’t have friends, don’t have anyone, don’t have anybody |
| Legal consequences | DUI, DWI, drink and drive, drugged driving, impaired driving, driving under (the) influence, driving while intoxicated/impaired/stoned/high/drunk/under influence, operating vehicle while impaired, OVI, underage drinking, drinking under 21, breathalyzer, jail, goal, prison, correctional facility, judicial system, detention, charges, accusation, smuggling, police, cops, detain, public citation, interdict, bail, legal consequence, misdemeanor, felon, expungement, probation, parole, court, judge, prosecutor, attorneys, lawyers, convict, verdict, habitual drunkard, criminal charge/record/case/law/offense/issue/trouble/problem/history, legal issue/trouble/problem, lost license, license revoked/suspended/cancelled, got in trouble with (the) law, lawsuit |
| Rehabilitation and treatment | rehab, rehabilitation, treatment, treatment center, recovery, recovery program, recovery center, detox |
| Coworkers | coworker, supervisor, employer, employee, staff, manager, boss, colleague, HR, human resource, work mate, work buddy, secretary, assistant, team lead, teammate, team member, client, customer |
| Physical health providers | Doctor, physician, nurse, clinician, healthcare provider, primary care provider, health provider, medical provider, surgeon, pharmacist, dentist, podiatrist, allergist, pulmonologist, ophthalmologist, otolaryngologist, (para)medics, pediatrician, endocrinologist, neurologist, rheumatologist, immunologist, nephrologist, gynecologist, urologist, anesthesiologist, dermatologist, cardiologist, obstetrician, radiologist, gastroenterologist, orthopedist, optometrist, medical specialist, medical practitioner, general practitioner, chiropractor |
| Others | teacher, lecturer, professor, spectator, school admin, others, someone else, other person/people, peer, roommate, roomie, flat mate, shop attendant, landlord, neighbor, pedestrian, hosts, guest, tourist, barista, waiter, waitress, owner, barber, server, worker, bartender, passenger, driver, onlooker, bystander, cashier, stranger, rental angent/agency, sales person/(wo)man, receptionist |
| Mental health services and providers | counselling, counsellor, therapy, therapist, psychiatrist, psychologist, psychotherapist, social worker, mental health provider/professional/specialist/practitioner |
| Community and support groups | support group, alcoholics anonymous, AA, narcotics anonymous, NA, marijuana anonymous, al anon, community, recovery meeting, self-help group, support network, therapy group, community center |
| Society | society, societal, publics, population, country, nation |
| Legalization | legalize, legalization, legality, legislation, legislative, legislature, legislator, regulation, regulatory, regulate, policy, recreational marijuana, legitimate, legitimacy |

Notes: We have performed pre-processing on the input texts to remove punctuations and convert to lowercase. We use regular expressions to handle derivations, inflections, different spellings, and edge cases. For example, for the “society” variable, we capture “nation” as a stand-alone word; we do not capture this string as part of another word, like “combination” and “imagination.”
