# Supplementary material for: Identifying Stigma Phenotypes in Social Media Narratives of Substance Use: Observational Study
Source: J Med Internet Res. 2025 Nov 13;27:e68695. doi: 10.2196/68695 (PMC12661227; doi:10.2196/68695)
Supplement: Multimedia Appendix 3 [file jmir_v27i1e68695_app3.docx]

Appendix 3. Full results of the Kruskal-Wallis and Dunn’s post-hoc tests

**Table S1. Individual level – loneliness and social isolation**

Kruskal-Wallis chi-squared = 369.07, df = 6, p-value < 2.2e-16

Comparison Z P.unadj P.adj

1 0 - 1 -11.31458489 1.111270e-29 2.333668e-28

2 0 - 2 0.45287570 6.506383e-01 1.000000e+00

3 1 - 2 12.12635308 7.658473e-34 1.608279e-32

4 0 - 3 2.76860288 5.629721e-03 1.182241e-01

5 1 - 3 13.68404820 1.264521e-42 2.655494e-41

6 2 - 3 2.40601436 1.612763e-02 3.386802e-01

7 0 - 4 -8.38718790 4.979089e-17 1.045609e-15

8 1 - 4 2.45703876 1.400876e-02 2.941839e-01

9 2 - 4 -9.06103267 1.292213e-19 2.713647e-18

10 3 - 4 -10.78793216 3.925220e-27 8.242963e-26

11 0 - 5 0.41908422 6.751546e-01 1.000000e+00

12 1 - 5 11.91913575 9.407939e-33 1.975667e-31

13 2 - 5 -0.02840581 9.773385e-01 1.000000e+00

14 3 - 5 -2.40235211 1.629002e-02 3.420904e-01

15 4 - 5 8.91363600 4.938577e-19 1.037101e-17

16 0 - 6 1.96862265 4.899644e-02 1.000000e+00

17 1 - 6 13.18681637 1.045062e-39 2.194631e-38

18 2 - 6 1.57571514 1.150915e-01 1.000000e+00

19 3 - 6 -0.84289837 3.992853e-01 1.000000e+00

20 4 - 6 10.21697082 1.664615e-24 3.495692e-23

21 5 - 6 1.58204818 1.136386e-01 1.000000e+00

**Table S2. Individual level – legal consequences**

Kruskal-Wallis chi-squared = 389.19, df = 6, p-value < 2.2e-16

Comparison Z P.unadj P.adj

1 0 - 1 -4.075690 4.587813e-05 9.634407e-04

2 0 - 2 5.375938 7.618497e-08 1.599884e-06

3 1 - 2 9.787782 1.270524e-22 2.668101e-21

4 0 - 3 -9.861783 6.095732e-23 1.280104e-21

5 1 - 3 -6.302271 2.933156e-10 6.159627e-09

6 2 - 3 -15.259354 1.426534e-52 2.995721e-51

7 0 - 4 1.766167 7.736777e-02 1.000000e+00

8 1 - 4 5.846556 5.018541e-09 1.053894e-07

9 2 - 4 -3.484741 4.926144e-04 1.034490e-02

10 3 - 4 11.434231 2.820237e-30 5.922499e-29

11 0 - 5 -1.642987 1.003856e-01 1.000000e+00

12 1 - 5 2.430358 1.508390e-02 3.167619e-01

13 2 - 5 -7.145052 8.996172e-13 1.889196e-11

14 3 - 5 8.413271 3.987308e-17 8.373346e-16

15 4 - 5 -3.410049 6.495124e-04 1.363976e-02

16 0 - 6 7.178199 7.063569e-13 1.483349e-11

17 1 - 6 11.454354 2.236238e-30 4.696099e-29

18 2 - 6 2.075224 3.796574e-02 7.972804e-01

19 3 - 6 16.629396 4.268784e-62 8.964447e-61

20 4 - 6 5.340825 9.252463e-08 1.943017e-06

21 5 - 6 8.894722 5.856587e-19 1.229883e-17

**Table S3. Individual level – rehabilitation and treatment**

Kruskal-Wallis chi-squared = 438.66, df = 6, p-value < 2.2e-16

Comparison Z P.unadj P.adj

1 0 - 1 9.21736245 3.044978e-20 6.394454e-19

2 0 - 2 14.82557007 1.001263e-49 2.102652e-48

3 1 - 2 5.91884650 3.242074e-09 6.808355e-08

4 0 - 3 15.34235724 3.984589e-53 8.367637e-52

5 1 - 3 7.05365117 1.742830e-12 3.659942e-11

6 2 - 3 1.54571605 1.221732e-01 1.000000e+00

7 0 - 4 14.30767812 1.959469e-46 4.114884e-45

8 1 - 4 5.74537995 9.171481e-09 1.926011e-07

9 2 - 4 0.08285016 9.339707e-01 1.000000e+00

10 3 - 4 -1.41080578 1.583019e-01 1.000000e+00

11 0 - 5 12.48268071 9.280850e-36 1.948979e-34

12 1 - 5 3.60856500 3.078954e-04 6.465803e-03

13 2 - 5 -2.20209793 2.765839e-02 5.808262e-01

14 3 - 5 -3.57744745 3.469659e-04 7.286285e-03

15 4 - 5 -2.19247504 2.834522e-02 5.952497e-01

16 0 - 6 18.07457420 5.054405e-73 1.061425e-71

17 1 - 6 9.65115256 4.860632e-22 1.020733e-20

18 2 - 6 3.95511450 7.649801e-05 1.606458e-03

19 3 - 6 2.20986898 2.711426e-02 5.693994e-01

20 4 - 6 3.72020983 1.990573e-04 4.180204e-03

21 5 - 6 6.01292563 1.822046e-09 3.826297e-08

**Table S4. Interpersonal level – co-workers**

Kruskal-Wallis chi-squared = 191.44, df = 6, p-value < 2.2e-16

Comparison Z P.unadj P.adj

1 0 - 1 -4.8969389 9.734108e-07 2.044163e-05

2 0 - 2 4.5893624 4.446021e-06 9.336643e-05

3 1 - 2 9.8159842 9.609562e-23 2.018008e-21

4 0 - 3 3.2067775 1.342308e-03 2.818846e-02

5 1 - 3 7.9998500 1.245709e-15 2.615989e-14

6 2 - 3 -1.1035473 2.697896e-01 1.000000e+00

7 0 - 4 0.6709049 5.022811e-01 1.000000e+00

8 1 - 4 5.5207076 3.376373e-08 7.090382e-07

9 2 - 4 -3.8344169 1.258625e-04 2.643113e-03

10 3 - 4 -2.5223451 1.165753e-02 2.448081e-01

11 0 - 5 0.9731963 3.304557e-01 1.000000e+00

12 1 - 5 5.9809275 2.218706e-09 4.659282e-08

13 2 - 5 -3.6556077 2.565734e-04 5.388042e-03

14 3 - 5 -2.3151798 2.060310e-02 4.326650e-01

15 4 - 5 0.2796427 7.797517e-01 1.000000e+00

16 0 - 6 7.4539673 9.057448e-14 1.902064e-12

17 1 - 6 12.5492060 4.015159e-36 8.431834e-35

18 2 - 6 3.1335765 1.726899e-03 3.626488e-02

19 3 - 6 3.9920436 6.550632e-05 1.375633e-03

20 4 - 6 6.6934775 2.179285e-11 4.576499e-10

21 5 - 6 6.5951568 4.248067e-11 8.920940e-10

**Table S5. Interpersonal level – Physical health care providers**

Kruskal-Wallis chi-squared = 264.02, df = 6, p-value < 2.2e-16

Comparison Z P.unadj P.adj

1 0 - 1 8.63956726 5.642634e-18 1.184953e-16

2 0 - 2 13.11829755 2.586809e-39 5.432299e-38

3 1 - 2 4.73902395 2.147501e-06 4.509753e-05

4 0 - 3 9.02979956 1.719865e-19 3.611717e-18

5 1 - 3 1.07717419 2.814025e-01 1.000000e+00

6 2 - 3 -3.29438623 9.863686e-04 2.071374e-02

7 0 - 4 9.30719697 1.312503e-20 2.756257e-19

8 1 - 4 1.13237208 2.574780e-01 1.000000e+00

9 2 - 4 -3.37046998 7.504008e-04 1.575842e-02

10 3 - 4 0.02081714 9.833915e-01 1.000000e+00

11 0 - 5 11.58906573 4.682117e-31 9.832446e-30

12 1 - 5 3.26694477 1.087149e-03 2.283013e-02

13 2 - 5 -1.38903440 1.648223e-01 1.000000e+00

14 3 - 5 1.95936828 5.006967e-02 1.000000e+00

15 4 - 5 1.99437248 4.611136e-02 9.683386e-01

16 0 - 6 14.36831119 8.180171e-47 1.717836e-45

17 1 - 6 6.37936417 1.778247e-10 3.734319e-09

18 2 - 6 1.83076374 6.713581e-02 1.000000e+00

19 3 - 6 4.88295646 1.045070e-06 2.194646e-05

20 4 - 6 4.99620756 5.846873e-07 1.227843e-05

21 5 - 6 3.13758776 1.703443e-03 3.577230e-02

**Table S6. Interpersonal level – Others**

Kruskal-Wallis chi-squared = 262.61, df = 6, p-value < 2.2e-16

Comparison Z P.unadj P.adj

1 0 - 1 -9.0630246 1.268824e-19 2.664531e-18

2 0 - 2 3.1949030 1.398778e-03 2.937435e-02

3 1 - 2 12.6577984 1.013006e-36 2.127312e-35

4 0 - 3 -0.8533121 3.934862e-01 1.000000e+00

5 1 - 3 7.7846840 6.988765e-15 1.467641e-13

6 2 - 3 -3.9329271 8.391769e-05 1.762272e-03

7 0 - 4 -5.7566310 8.580918e-09 1.801993e-07

8 1 - 4 2.9646086 3.030683e-03 6.364434e-02

9 2 - 4 -9.0618197 1.282921e-19 2.694134e-18

10 3 - 4 -4.6853355 2.795017e-06 5.869536e-05

11 0 - 5 -3.2693503 1.077948e-03 2.263690e-02

12 1 - 5 5.8032686 6.503447e-09 1.365724e-07

13 2 - 5 -6.6050612 3.973532e-11 8.344417e-10

14 3 - 5 -2.2684048 2.330455e-02 4.893955e-01

15 4 - 5 2.6106502 9.037027e-03 1.897776e-01

16 0 - 6 3.4682429 5.238735e-04 1.100134e-02

17 1 - 6 12.5228197 5.600944e-36 1.176198e-34

18 2 - 6 0.4139361 6.789209e-01 1.000000e+00

19 3 - 6 4.1729769 3.006454e-05 6.313554e-04

20 4 - 6 9.1015736 8.903236e-20 1.869680e-18

21 5 - 6 6.7379895 1.605931e-11 3.372455e-10

**Table S7. Behavior settings – mental health services and providers**

Kruskal-Wallis chi-squared = 211.89, df = 6, p-value < 2.2e-16

Comparison Z P.unadj P.adj

1 0 - 1 -2.2852290 2.229940e-02 4.682874e-01

2 0 - 2 6.1106937 9.919900e-10 2.083179e-08

3 1 - 2 8.7072878 3.112322e-18 6.535876e-17

4 0 - 3 7.8736459 3.444530e-15 7.233513e-14

5 1 - 3 10.3291027 5.204514e-25 1.092948e-23

6 2 - 3 2.2267123 2.596651e-02 5.452966e-01

7 0 - 4 0.2088128 8.345944e-01 1.000000e+00

8 1 - 4 2.4682522 1.357746e-02 2.851267e-01

9 2 - 4 -5.8085456 6.301788e-09 1.323375e-07

10 3 - 4 -7.5744775 3.605760e-14 7.572096e-13

11 0 - 5 5.6474298 1.628643e-08 3.420151e-07

12 1 - 5 8.1845493 2.733247e-16 5.739819e-15

13 2 - 5 -0.3908047 6.959416e-01 1.000000e+00

14 3 - 5 -2.5628408 1.038196e-02 2.180212e-01

15 4 - 5 5.3569227 8.465130e-08 1.777677e-06

16 0 - 6 7.4397038 1.009113e-13 2.119137e-12

17 1 - 6 9.9614842 2.246831e-23 4.718345e-22

18 2 - 6 1.6197746 1.052807e-01 1.000000e+00

19 3 - 6 -0.6291029 5.292817e-01 1.000000e+00

20 4 - 6 7.1352804 9.659007e-13 2.028391e-11

21 5 - 6 1.9728055 4.851772e-02 1.000000e+00

**Table S8. Community level – community and support groups**

Kruskal-Wallis chi-squared = 196.33, df = 6, p-value < 2.2e-16

Comparison Z P.unadj P.adj

1 0 - 1 -7.4380193 1.022061e-13 2.146328e-12

2 0 - 2 0.7705465 4.409758e-01 1.000000e+00

3 1 - 2 8.4632607 2.600037e-17 5.460078e-16

4 0 - 3 4.1899540 2.790110e-05 5.859230e-04

5 1 - 3 11.4468599 2.438185e-30 5.120188e-29

6 2 - 3 3.5597021 3.712757e-04 7.796790e-03

7 0 - 4 0.9569976 3.385684e-01 1.000000e+00

8 1 - 4 8.3211643 8.710341e-17 1.829172e-15

9 2 - 4 0.2234609 8.231768e-01 1.000000e+00

10 3 - 4 -3.2184751 1.288741e-03 2.706357e-02

11 0 - 5 -1.1807967 2.376835e-01 1.000000e+00

12 1 - 5 6.3230088 2.565187e-10 5.386892e-09

13 2 - 5 -1.9965697 4.587195e-02 9.633110e-01

14 3 - 5 -5.3747876 7.667299e-08 1.610133e-06

15 4 - 5 -2.1342209 3.282470e-02 6.893187e-01

16 0 - 6 4.4253722 9.627613e-06 2.021799e-04

17 1 - 6 11.9138634 1.002235e-32 2.104694e-31

18 2 - 6 3.7860232 1.530774e-04 3.214626e-03

19 3 - 6 0.1130465 9.099937e-01 1.000000e+00

20 4 - 6 3.4227023 6.200195e-04 1.302041e-02

21 5 - 6 5.6491208 1.612705e-08 3.386680e-07

**Table S9. Societal level – society**

Kruskal-Wallis chi-squared = 104.34, df = 6, p-value < 2.2e-16

Comparison Z P.unadj P.adj

1 0 - 1 -2.9491385 3.186611e-03 6.691883e-02

2 0 - 2 3.6958332 2.191669e-04 4.602504e-03

3 1 - 2 6.8805049 5.964079e-12 1.252457e-10

4 0 - 3 -4.2792729 1.875048e-05 3.937601e-04

5 1 - 3 -1.6056713 1.083461e-01 1.000000e+00

6 2 - 3 -7.9259902 2.263355e-15 4.753045e-14

7 0 - 4 -3.0096084 2.615847e-03 5.493278e-02

8 1 - 4 -0.2130211 8.313105e-01 1.000000e+00

9 2 - 4 -6.7340390 1.650169e-11 3.465355e-10

10 3 - 4 1.3356015 1.816796e-01 1.000000e+00

11 0 - 5 0.2384723 8.115148e-01 1.000000e+00

12 1 - 5 3.2409344 1.191386e-03 2.501911e-02

13 2 - 5 -3.5047825 4.569805e-04 9.596590e-03

14 3 - 5 4.5623373 5.058730e-06 1.062333e-04

15 4 - 5 3.2853762 1.018463e-03 2.138772e-02

16 0 - 6 2.1180569 3.417025e-02 7.175752e-01

17 1 - 6 5.1004196 3.389013e-07 7.116928e-06

18 2 - 6 -1.4664780 1.425181e-01 1.000000e+00

19 3 - 6 6.2587440 3.880903e-10 8.149896e-09

20 4 - 6 5.0591704 4.210844e-07 8.842772e-06

21 5 - 6 1.9115473 5.593428e-02 1.000000e+00

**Table S10. Societal level – legalization**

Kruskal-Wallis chi-squared = 103.34, df = 6, p-value < 2.2e-16

Comparison Z P.unadj P.adj

1 0 - 1 2.48261121 1.304234e-02 2.738890e-01

2 0 - 2 3.61657187 2.985305e-04 6.269140e-03

3 1 - 2 1.20268430 2.290985e-01 1.000000e+00

4 0 - 3 -5.32462968 1.011589e-07 2.124337e-06

5 1 - 3 -7.88143323 3.236470e-15 6.796588e-14

6 2 - 3 -8.92223867 4.569562e-19 9.596080e-18

7 0 - 4 -1.29130805 1.965969e-01 1.000000e+00

8 1 - 4 -3.78461939 1.539440e-04 3.232824e-03

9 2 - 4 -4.89101295 1.003184e-06 2.106686e-05

10 3 - 4 4.01790385 5.871815e-05 1.233081e-03

11 0 - 5 1.68295452 9.238390e-02 1.000000e+00

12 1 - 5 -0.77193296 4.401541e-01 1.000000e+00

13 2 - 5 -1.93839065 5.257558e-02 1.000000e+00

14 3 - 5 7.00508533 2.468354e-12 5.183543e-11

15 4 - 5 2.96818849 2.995606e-03 6.290772e-02

16 0 - 6 1.67905930 9.314049e-02 1.000000e+00

17 1 - 6 -0.70565510 4.804027e-01 1.000000e+00

18 2 - 6 -1.83927035 6.587543e-02 1.000000e+00

19 3 - 6 6.86922487 6.455168e-12 1.355585e-10

20 4 - 6 2.93078881 3.381025e-03 7.100154e-02

21 5 - 6 0.04264475 9.659847e-01 1.000000e+00
